# Supplementary material for: Environmental stability and phenotypic plasticity benefit the cold-water coral Desmophyllum dianthus in an acidified fjord
Source: Commun Biol. 2022 Jul 9;5:683. doi: 10.1038/s42003-022-03622-3 (PMC9271058; doi:10.1038/s42003-022-03622-3)
Supplement: Supplementary file 2 — Supplementary Material [file 42003_2022_3622_MOESM2_ESM.pdf]

Supplementary material to:

Environmental stability and phenotypic plasticity benefit the cold-water coral *Desmophyllum dianthus* in an acidified fjord

Kristina K. Beck[1,2*], Gertraud M. Schmidt-Grieb[1], Jürgen Laudien[1], Günter Försterra[3,4], Verena Häussermann[3,5], Humberto E. González[6,7], Juan Pablo Espinoza[3,4], Claudio Richter[1,2 #], Marlene Wall[1,8 #]

[1]Alfred Wegener Institute Helmholtz Centre for Polar and Marine Research, Bremerhaven, Germany

[2]University of Bremen, Bremen, Germany

[3]Fundación San Ignacio del Huinay, Puerto Montt, Chile

[4]Pontificia Universidad Católica de Valparaíso, Valparaíso, Chile

[5]Universidad San Sebastián, Puerto Montt, Chile

[6]Universidad Austral de Chile, Valdivia and Punta Arenas, Chile

[7]Centro FONDAP de Investigación en Dinámica de Ecosistemas Marinos de Altas Latitudes (IDEAL), Valdivia, Chile

[8]GEOMAR, Kiel, Germany

* Corresponding author: Kristina.Beck@awi.de

[#] These authors jointly supervised this work

Supplementary Results

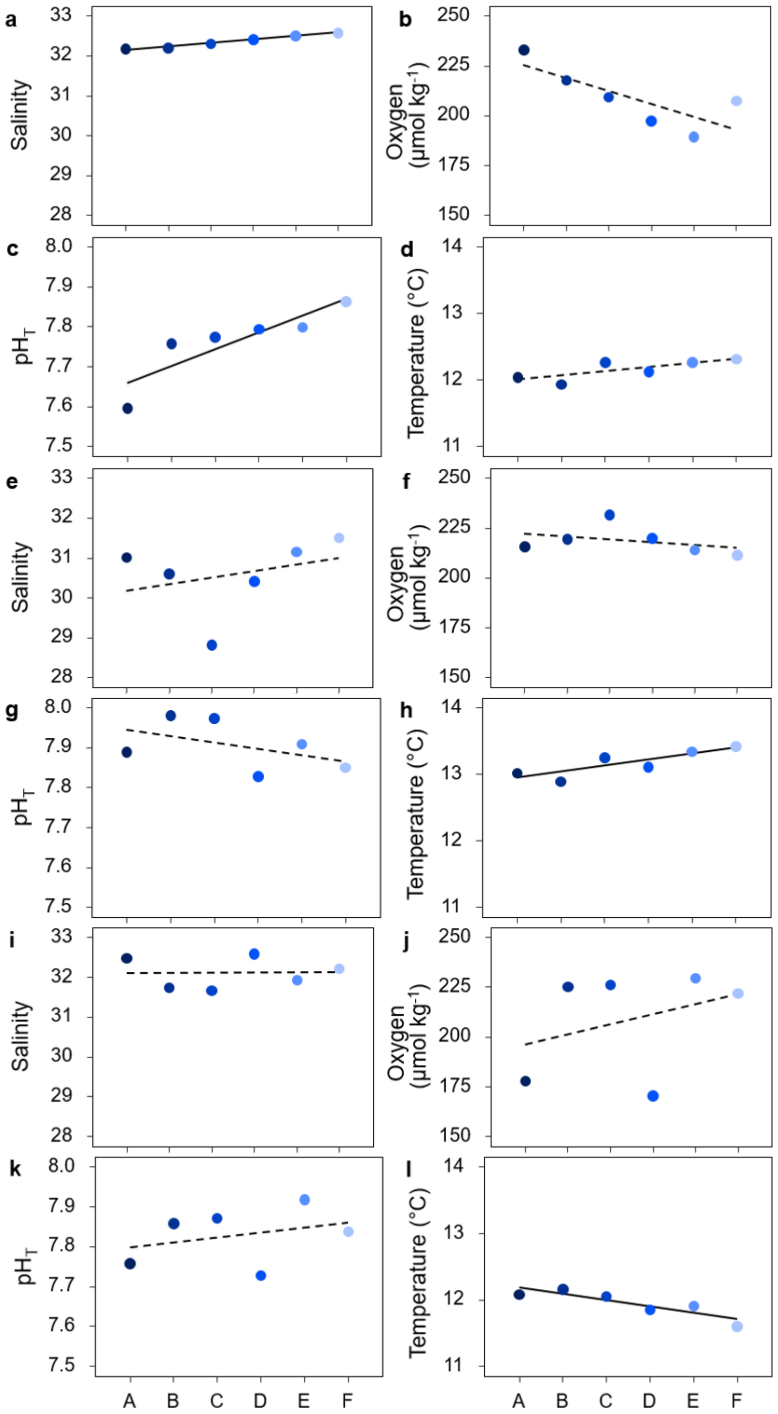

Supplementary Figure 1: Seasonal horizontal gradients at 20 m depth of Comau Fjord, Chile. Water parameters at six coral stations at 20 m water depth along the fjord from head to mouth (A-F, colours correspond to Fig. 1) in austral summer (January, a-d), autumn (May, e-h) and winter (August, i-l). Salinity and oxygen concentration were measured once during each season with a CTD. Total pH (pH_T) was calculated from water samples once during each season and calculated from total alkalinity (TA) and dissolved inorganic carbon (DIC) using CO2SYS[1]. Temperature was measured every 15 min. over a period of three to four months for each season using TidbiT temperature loggers and the mean temperature was plotted. Regression lines with significant p-values ($p < 0.05$) are plotted as solid lines, non-significant results are plotted as dotted lines.

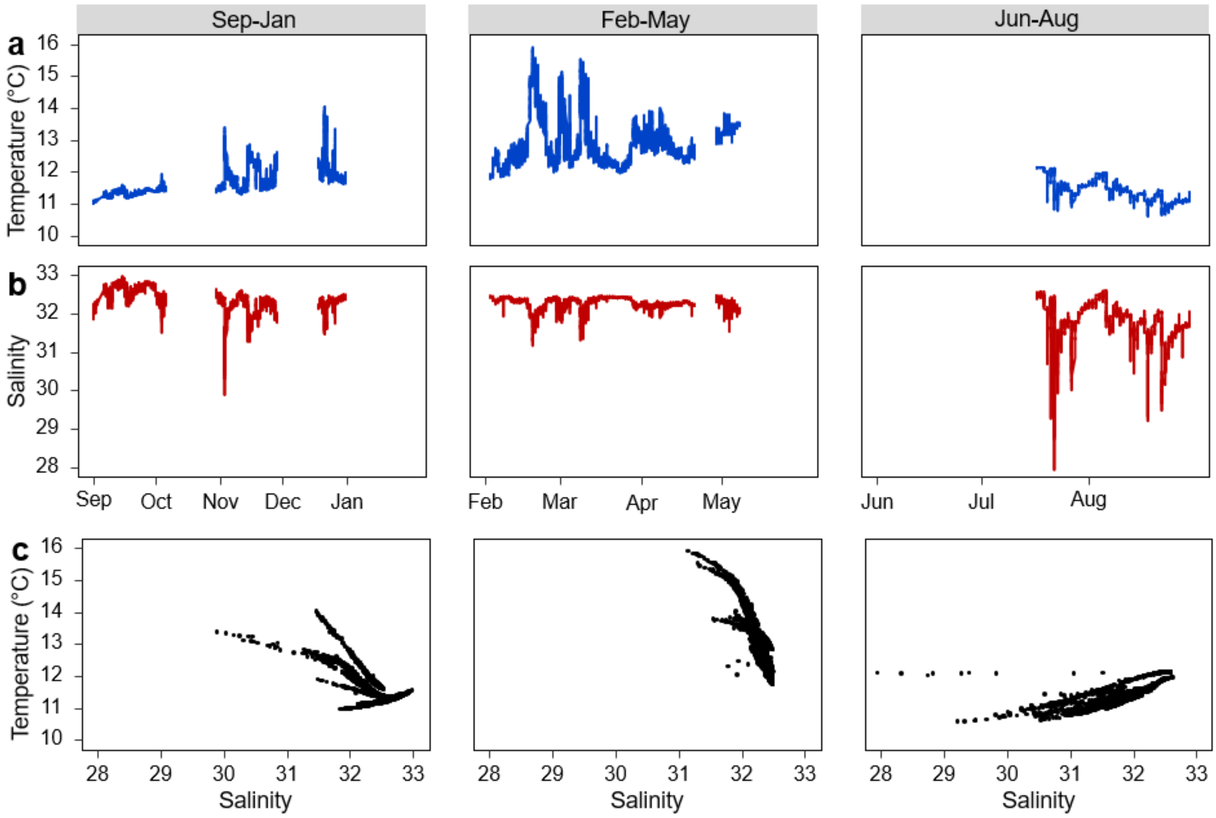

Supplementary Figure 2: Seasonal water temperature and salinity of Comau Fjord, Chile. a) Water temperature (blue) and b) salinity (red) were measured with a CTD installed at 25 m depth at station X between September 2016 and August 2017. c) Correlation between temperature and salinity.

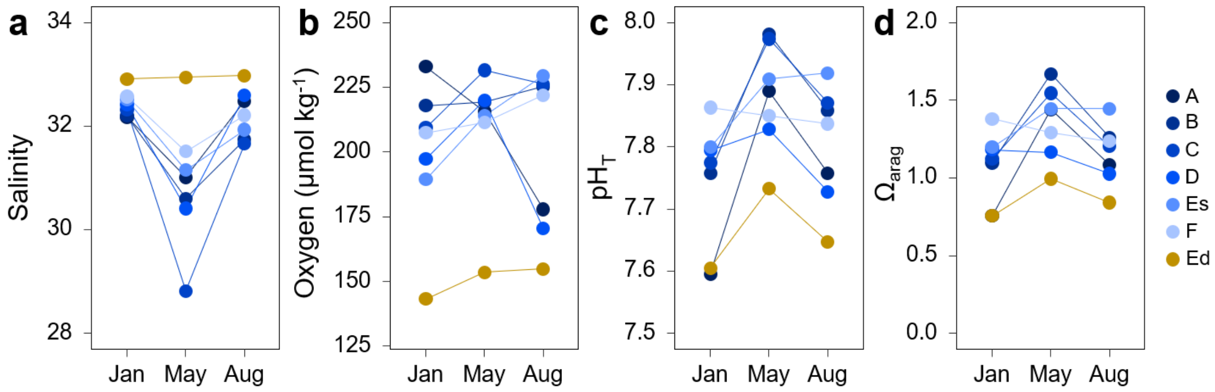

Supplementary Figure 3: Seasonal environmental conditions at shallow and deep coral stations in Comau Fjord, Chile. a) Salinity and b) oxygen from CTD, c) seawater pH_T and d) aragonite saturation (Ω_{arag}) calculated from TA and DIC in austral summer (January), autumn (May) and winter (August). Single data points for all shallow stations at 20 m water depth (A-F) are shown in blue and conditions at the deep station at 300 m water depth (Ed) in yellow.

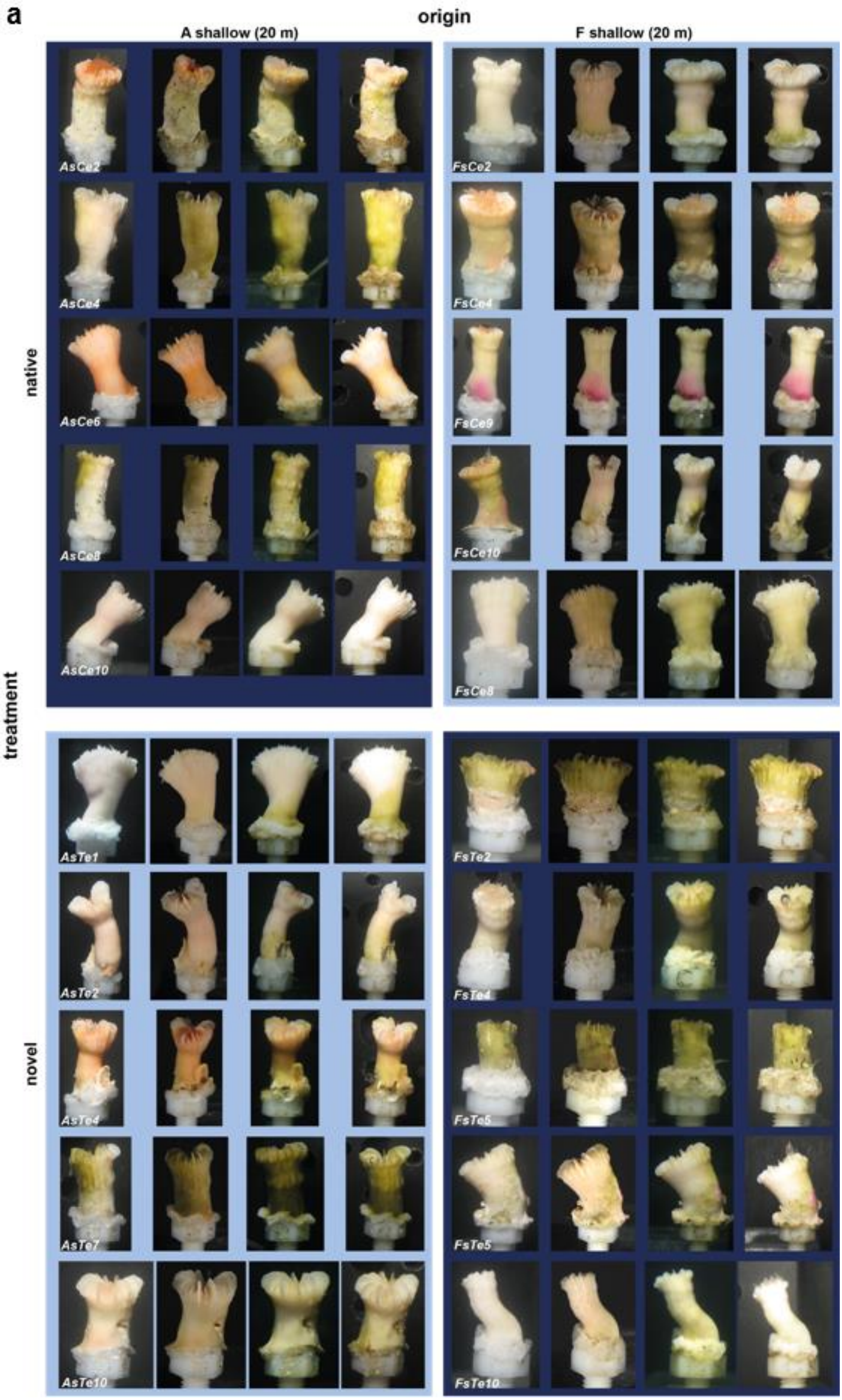
a
origin
A shallow (20 m)
F shallow (20 m)
treatment
native
novel
AsCe2
AsCe4
AsCe6
AsCe8
AsCe10
FsCe2
FsCe4
FsCe9
FsCe10
FsCe8
AsTe1
AsTe2
AsTe4
AsTe7
AsTe10
FsTe2
FsTe4
FsTe5
FsTe5
FsTe10

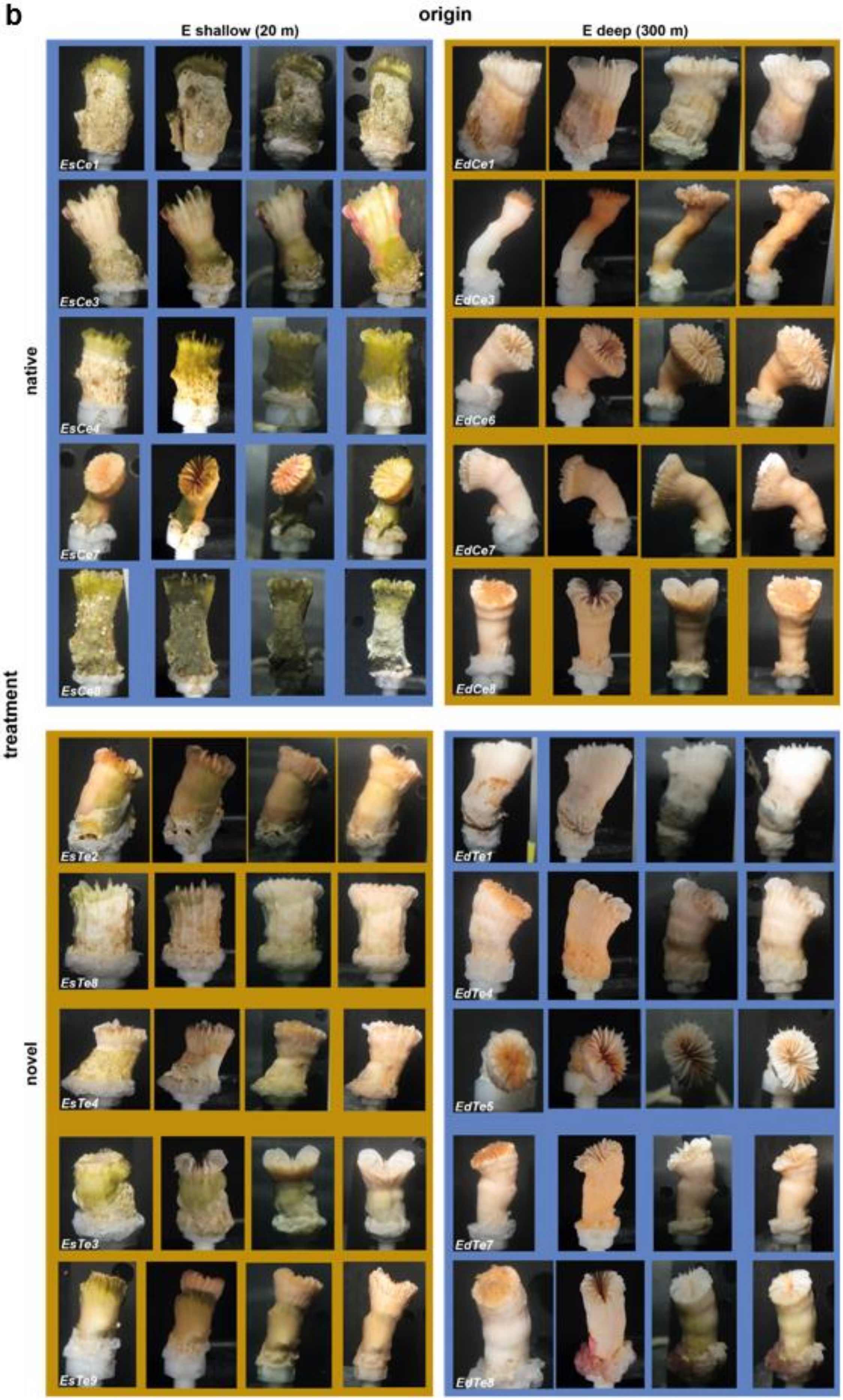
b
origin
E shallow (20 m)
E deep (300 m)
treatment
native
novel
EsCe1
EsCe3
EsCe4
EsCe7
EsCe8
EdCe1
EdCe3
EdCe6
EdCe7
EdCe8
EsTe2
EsTe8
EsTe4
EsTe3
EsTe9
EdTe1
EdTe4
EdTe5
EdTe7
EdTe8

Supplementary Figure 4: Pictures of experimental individuals of *Desmophyllum dianthus* throughout the reciprocal transplantation experiment. Pictures of 5 of the 8-10 native and novel corals from each reciprocal transplantation station at a) shallow (20 m depth; station A: fjord head, station F: fjord mouth) and b) between shallow (20 m; station Es) and deep (300 m depth; station Ed) are displayed at the four sampling dates: September 2016, January, May and August 2017. Background colour indicates the station of origin, e.g. the background colour of the novel corals at station Es is the colour of the deep station. The labels of the corals indicate: 1) station of origin (A, E, F), 2) depth of origin (s = shallow, d = deep), 3) native (C) or novel (T), 4) experimental coral (e) and 5) replicate number. Note the expansion of tissue area of the novel corals at station Ed.

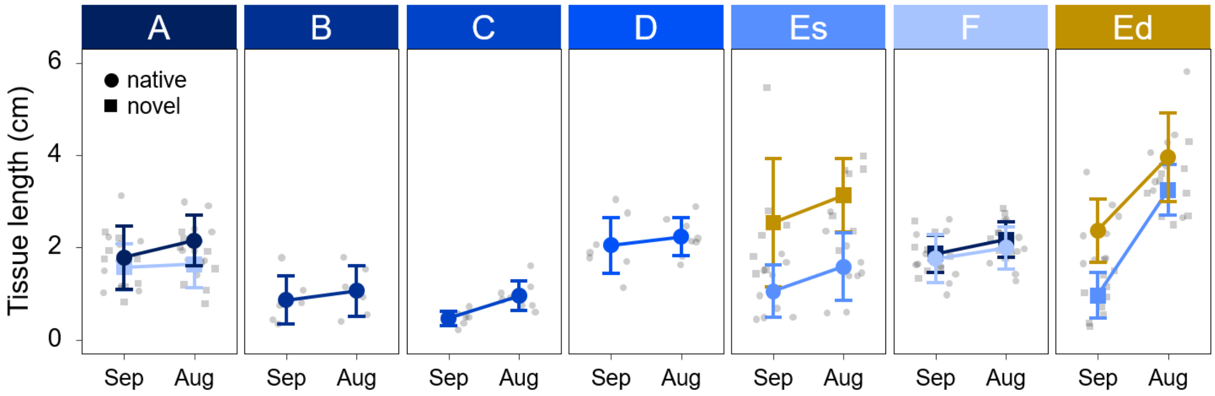

Supplementary Figure 5: Seasonal tissue coverage of native and novel *Desmophyllum dianthus* in Comau Fjord, Chile. The length of the tissue covered calyx of *D. dianthus* (mean ± standard deviation) at six stations at 20 m water depth along the fjord from head to mouth (A-F) is shown in blue and at one station at 300 m water depth (Ed) in yellow (data points in grey). Native corals (circles) were re-installed at the same station after collection in September 2016 and novel corals (squares) were cross-transplanted between the shallow stations at the head (A) and the mouth of the fjord (F) and between shallow (Es) and deep (Ed). Tissue length was measured at the beginning (September 2017) and end (August 2017) of the reciprocal transplantation experiment using the same individuals in each season (N = 6-10 independent samples). Note: As the tissue covered surface area changed only slightly at the shallow stations between September 2016 and August 2017, the surface area measurements carried out with a digital calliper at the end of the experiment (August 2017) were used as variable of reference for all sampling dates (January, May and August 2017). As the tissue covered surface area of native and novel corals at station Ed increased largely over time, the surface area of the corals at Ed was measured for each sampling date using scaled pictures of the corals.

Biomass

In general, the tissue biomass of native and novel corals at 300 m depth was higher than that of all corals at 20 m water depth (p-value < 0.001, Supplementary Figures 6, Supplementary Data 2). Transplantation of corals from 20 m to 300 m water depth had a significant effect on the tissue biomass of novel corals. Not only was the biomass of novel corals at 300 m depth higher than the biomass of native corals at this station (p-value < 0.001, Supplementary Figures 6, Supplementary Data 2), but the corals were also able to expand the tissue covered surface area (Supplementary Figures 4 and 5).

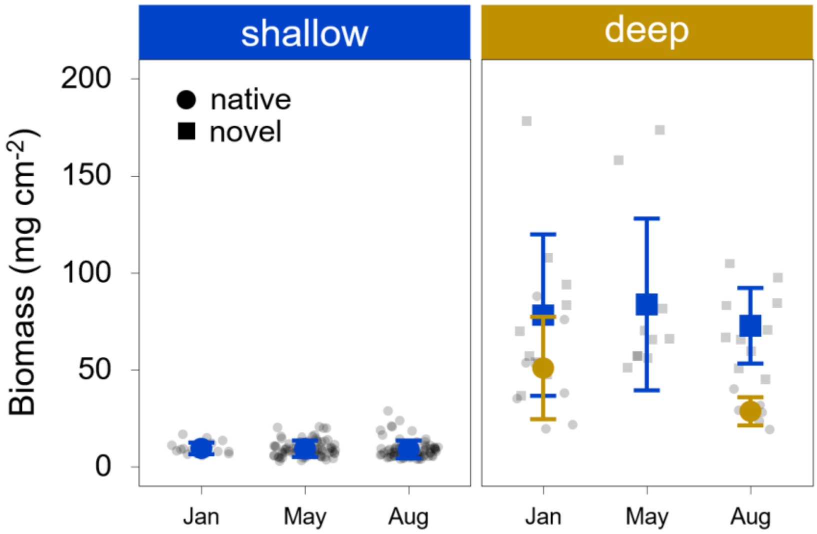

Supplementary Figure 6: Seasonal tissue biomass of native and novel *Desmophyllum dianthus* of Comau Fjord, Chile. Tissue biomass of *D. dianthus* (mean ± standard deviation) at all six shallow stations (20 m depth, A-F) is shown in blue and at one deep station (300 m depth, Ed) in yellow, with native corals shown as circles and novel corals shown as squares. Note that there are no novel corals at the shallow stations. Corals were sampled after four, eight and eleven months (January, May and August 2017) to determine their tissue biomass (N = 6-76 independent samples).

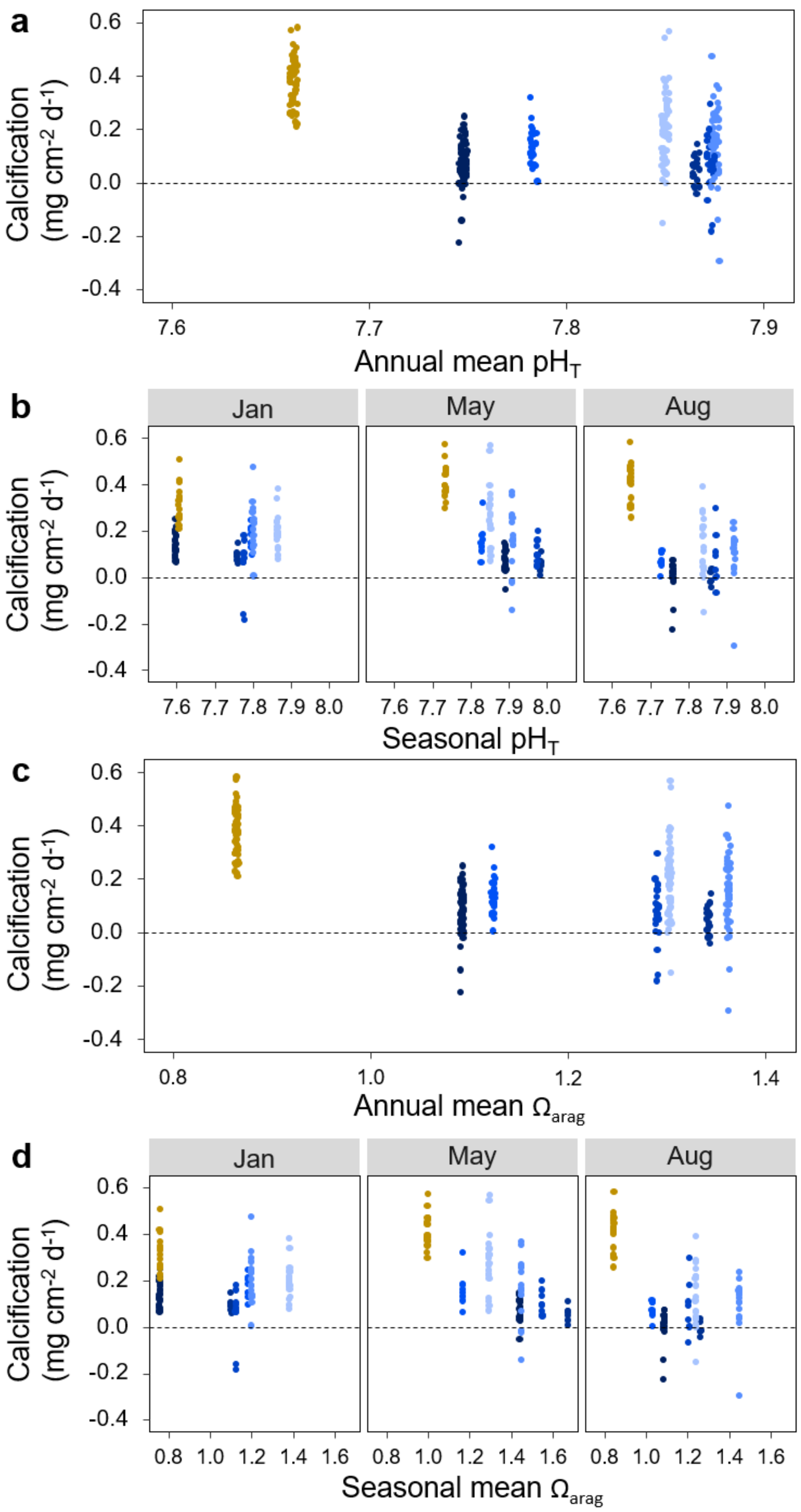
a
b
c
d
Calcification
(mg cm-2 d-1)
Annual mean pHT
Jan
May
Aug
Seasonal pHT
Annual mean Ωarag
Seasonal mean Ωarag
0.6
0.4
0.2
0.0
-0.2
-0.4
7.6
7.7
7.8
7.9
8.0
0.8
1.0
1.2
1.4
1.6

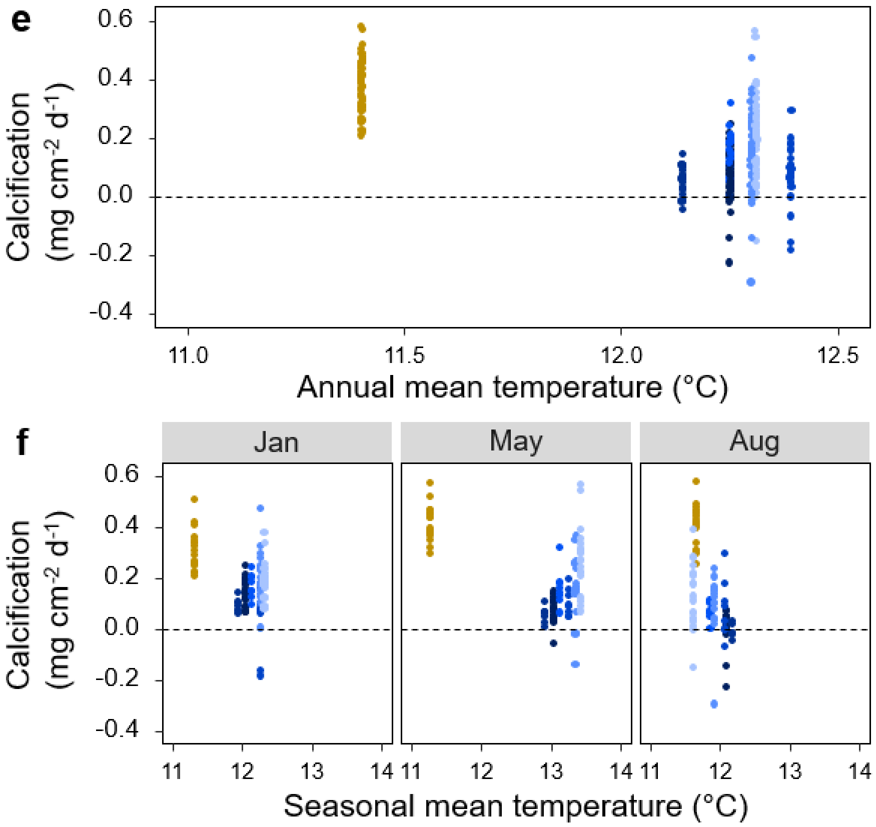

Supplementary Figure 7: Relationship between calcification rates of *Desmophyllum dianthus*, seawater pH, aragonite saturation and temperature at sampling stations in Comau Fjord, Chile. Combined calcification data of all three seasons (N = 19-57 independent samples) plotted against a) annual mean seawater pH_T, b) seasonal pH_T, c) annual mean aragonite saturation (Ω_{arag}), d) seasonal Ω_{arag}, e) annual mean temperature and f) seasonal mean temperature. Calcification rates of *D. dianthus* at six stations at 20 m water depth along the fjord (A-F) are shown in blue and calcification rates at one station at 300 m water depth (Ed) in yellow. Calcification rates of native and novel corals were measured after four, eight and eleven months (January, May and August 2017) using the same individuals in each season. Note that native and novel corals at each station are combined in this graph.

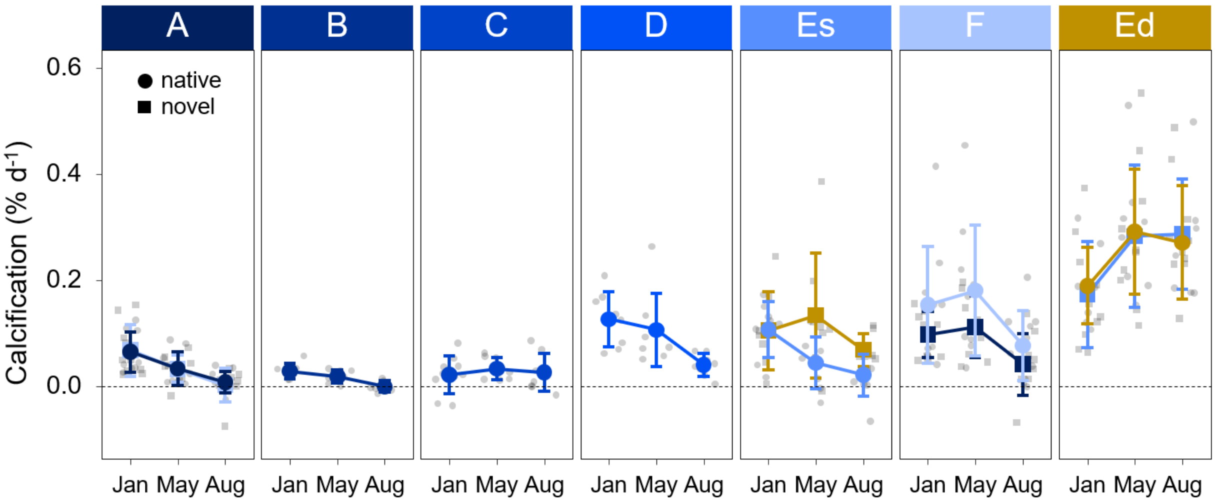

Supplementary Figure 8: Calcification rates of native and novel *Desmophyllum dianthus* in Comau Fjord, Chile. Calcification rates of *D. dianthus* (mean ± standard deviation) at six stations at 20 m water depth along the fjord from head to mouth (A-F) are shown in blue and at one station at 300 m water depth (Ed) in yellow (data points in grey). Native corals (circles) were re-installed at the same station after collection in September 2016 and novel corals (squares) were cross-transplanted between the shallow stations at the head (A) and the mouth of the fjord (F) and between shallow (Es) and deep (Ed). Calcification rates were measured after four, eight and eleven months (January, May and August 2017) using the same individuals in each season (N= 6-10 independent samples) and are standardized to the skeletal dry mass of the corals at the beginning of the respective growth period.

Supplementary Table 1: Linear mixed effect models for calcification and respiration rates of *Desmophyllum dianthus*. Only relevant results are displayed here. Significant p-values are shown in bold.

Fixed effects	Sum Sq	Mean Sq	NumDF	DenDF	F value	Pr(>F)
Calcification (model 1: shallow stations)						
**season**	0.254	0.127	2	96.308	19.800	**<0.001**
**station**	0.233	0.047	5	43.578	7.257	**<0.001**
Calcification (model 2: stations A, F, Es, Ed)						
**station**	1.401	0.467	3	64.601	51.073	**<0.001**
**transplant**	0.012	0.012	1	64.664	1.307	0.257
**station*transplant**	0.069	0.023	3	64.601	2.512	0.066
Respiration (model 1: shallow stations)						
**season**	32.414	16.207	2	96.108	5.489	**0.006**
**station**	143.820	28.764	5	44.210	9.742	**<0.001**
Respiration (model 2: stations A, F, Es, Ed)						
**station**	712.12	237.375	3	202	65.207	**<0.001**
**transplant**	1.23	1.227	1	202	0.337	0.562
**station*transplant**	26.82	8.941	3	202	2.456	0.064

Supplementary Discussion

The respiration rates of deep corals are also influenced by the higher incubation temperatures compared to the *in situ* temperature at the deep station. Due to the standardisation of respiration measurements for comparability and because we do not know the temperature performance curves of *D. dianthus*, we can only speculate if the change in temperature led to an under- or overestimation of the respiration rates of deep corals. As we expect the deep corals to have higher mitochondria density and activity (based on increased calcification rates, higher biomass as well as expected enhanced nutrition), this should result in elevated respiration rates under similar temperatures. Thus, by using the same temperatures, we gain insights into the metabolic potential rather than the exact *in situ* respiration rate. In addition, the effect of a 2 °C temperature change from 11.8 °C in winter to 14.2 °C in summer is evident in the native shallow corals at station Es, where the respiration rate increases from 1.7 µmol cm^{-2} d^{-1} in winter to a maximum of 4.2 µmol cm^{-2} d^{-1} in summer. This suggests that an increase of about 3°C (from 11.3 °C *in situ* to 14.2 °C in incubations in summer) could lead to a respiration rate of approx. 5 µmol cm^{-2} d^{-1} but not to 8 µmol cm^{-2} d^{-1} as measured for the deep corals in summer. The generally twice as high metabolic rate of deep corals over the seasons is therefore rather an indication of a changed metabolism in deep corals.

Supplementary Methods

Coral physiology

The tissue covered surface area of the experimental corals was used as reference variable for calcification (mg cm^{-2} d^{-1}) and respiration rates (µmol cm^{-2} d^{-1}). The outer surface area of the coral skeletons was measured at the end of the field study (August 2017) using a digital calliper (reading to 0.01 mm). A modulated formula for a truncated cone after the geometric approximation “Advanced Geometry” by Naumann et al.[2] was used to calculate the surface area based on the trumpet shape of *D. dianthus*. For this, the shape of the coral was approximated to a cup, the surface areas of the individual septa were not considered.

The surface area of *D. dianthus* has previously been measured as the outer surface area of the coral polyp, including its oral side[3,4]. A conceptual problem with this approach is the cup shape of *D. dianthus* as the oral side of the calyx is not tissue covered but the tissue coats the

skeleton on the inside of the calyx. A more straightforward approach is to consider all parts of the coral tissue that are in direct contact with the skeleton, assuming that these contribute to calcification and respiration of the polyp. This includes the tissue covered areas on the outside (but excluding the oral side of the polyp; A_o) and inside (tissue covered surface area of the coelenteron; A_i) of the calyx. For this purpose, the inner and outer tissue covered surface areas of 30 *D. dianthus* specimen were measured with a digital calliper and surface area I (outer and oral part of the polyp)[3,4] and surface area II (inner and outer surface areas of the calyx excluding the oral side of the polyp) were calculated and correlated (Supplementary Figure 9, regression line: Surface area II = 1.319 x Surface area I, R^2 = 0.996, n = 30). In case of the surface area I (A), the following formula was applied:

$$A\ (\mathrm{cm}^2) = \pi \times [R_o^2 + s_o \times (r_o + R_o)] \quad (1)$$

where R_o and r_o are the outer oral and aboral radius (half diameter D_o and d_o) of the polyp, respectively, and s_o is the outer slant height calculated from

$$s_o = \sqrt{(R_o - r_o)^2 + h_o^2} \quad (2)$$

where h_o is the mean height of the outer tissue covered surface area of the polyp (Supplementary Figure 10a). The surface area II (A_i and A_o) were calculated as:

$$A_i\ (\mathrm{cm}^2) = \pi \times [r_i^2 \times s_i \times (r_i + R_i)] \quad (3)$$

where R_i and r_i are the oral and aboral radius (half diameter D_i and d_i) of the inner tissue-coated surface area of the calyx (coelenteron), respectively, and s_i is the inner slant height of the calyx (calculated from the measured inner height h_i, Supplementary Figure 10b); and

$$A_o\ (\mathrm{cm}^2) = \pi \times [s_o \times (r_o + R_o)] \quad (4)$$

where R_o and r_o are the oral and aboral radius of the outer tissue covered surface area of the calyx, respectively, and s_o is the outer slant height of the calyx (calculated from the measured outer height h_o, Supplementary Figure 10a). Due to the elliptical shape of the calyx of *D. dianthus*, the inner and outer diameters of the oral and aboral side of the polyp were each measured twice and the mean diameter was used to calculate the oral and aboral radius for both surface areas (I and II), respectively (Supplementary Figure 10c).

Using this correlation of surface areas I and II (Surface area II = 1.319 × Surface area I), the tissue covered surface area II can be calculated from outer measurements of the polyp (surface area I).

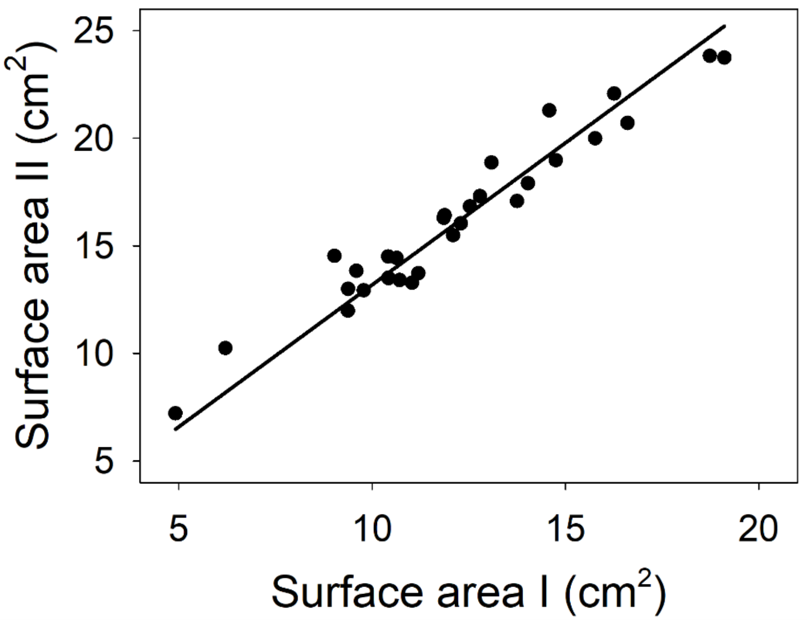

Supplementary Figure 9: Surface area measurements of *Desmophyllum dianthus*. Scatter plot showing the linear regression of the surface areas of I) the outer surface area of the coral polyp (including the oral side of the polyp) and II) the inner (tissue coated surface area of the coelenteron) and outer (excluding the oral side of the polyp) tissue covered surface area of the calyx (regression line: Surface area II = 1.319 × Surface area I, R^2 = 0.996, n = 30). Black dots represent individual specimens.

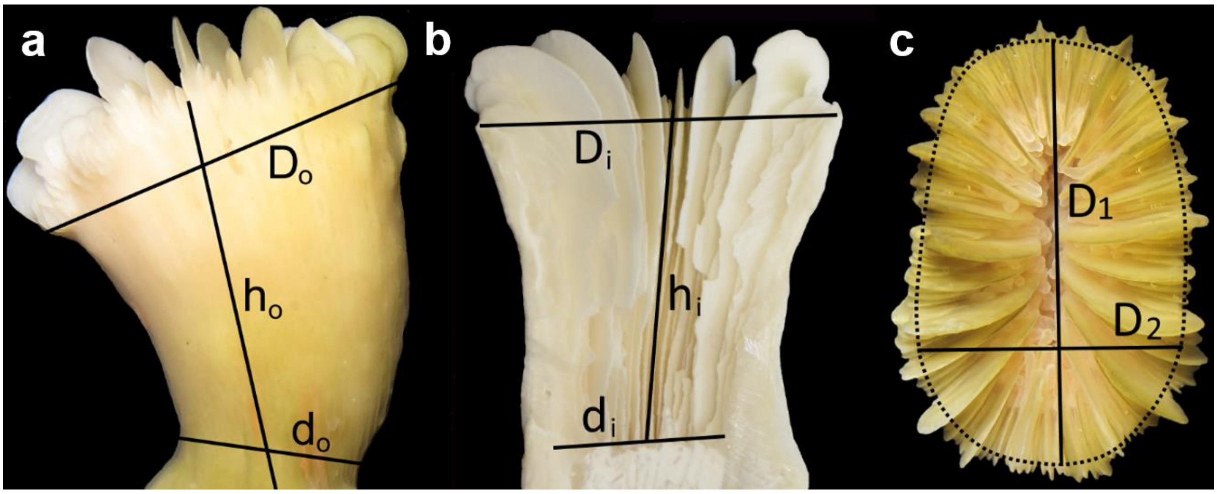

Supplementary Figure 10: Measurements of the tissue covered surface area of *Desmophyllum dianthus*. Measurements of the a) outer surface area, b) inner surface area, and c) diameters at the oral side. Dashed line = outer edge of calyx wall (excluding septa), D = oral diameter, d = aboral diameter, h = height, X_i = inner diameter/height, X_o = outer diameter/height.

In order to determine if the tissue covered surface area of an individual varied over the duration of the experiment and whether the surface area measurements of the shallow corals at the end of the experiment (August 2017) were representative for the whole duration of the experiment, scaled pictures of the same side of each individual from September 2016 and August 2017 were used and analysed using the software ImageJ. The extent of the tissue along the calyx was measured from the oral to the aboral edge of the tissue at three points on this image and then averaged. The tissue length and the ability to extend or retract the tissue is also an important parameter for changes in coral tissue biomass.

In addition to the experimental corals, 30 individuals of *D. dianthus* were collected at each of the shallow stations and eight individuals at the deep station in September 2016 and prepared as described in the main text by fixing them on screws. For cross-transplantation, another 30 corals were sampled at the shallow stations A, F and Es and eight corals at Ed. This gives a total of 188 native corals along the horizontal gradient and at depth and 98 novel corals at shallow and deep stations (tissue corals). The size of tissue corals (oral diameter: 14.96 ± 3.94 mm) was representative for the population.

At the end of each season (January, May and August 2017), ten native corals were collected at each of the shallow stations. In addition, ten novel corals were collected at stations A, F and Ed. Only in austral summer (January), eight native corals were collected at station Ed and eight novel corals at station Es. After collection, corals were transported to the research station, where they were unscrewed from the plates and maintained in flow-through aquaria for a maximum of four days before being removed from the screws, snap-frozen in liquid nitrogen, transported to the Alfred Wegener Institute (AWI) within liquid nitrogen containers (dry shipper) and stored at -80 °C until processing. Unfortunately, most frozen coral samples collected in austral summer (except for native and novel corals from stations Ed and F) were lost by malfunction of one of the freezers, so that we had to discard the thawed samples from tissue analyses.

Frozen tissue corals from station Ed were cut in half using a diamond-tipped saw (FKS/E, Proxxon S.A., Wecker, Luxemburg) in a -30 °C cold room to prevent the tissue from thawing during treatment. Only one half of each coral specimen of station Ed was used for biomass determination, while whole corals were used from all shallow stations. The tissue was separated from the skeleton working on ice and using an airbrush (Starter Class set, Revell GmbH, Bünde, Germany) connected to pressurised air at 5 bar and the tissue slurry was

homogenized using an Ultra Turrax (T18 basic, IKA GmbH & Co. KG, Staufen, Germany). A subsample (1 mL) of the tissue slurry was taken on a pre-combusted (4 hours at 500°C) and pre-weighed filter (GF/C; Whatman, GF Healthcare Life Sciences, Amersham, United Kingdom) for the determination of tissue biomass (dry mass). The filter with the tissue sample was dried to constant mass (24 hours at 60 °C) and weighed again using an electronic fine balance (Sartorius M2P, Sartorius AG, Göttingen, Germany; precision: 0.001 mg).

The surface area covered by tissue of the frozen tissue corals was measured using a digital calliper (as described above) prior to the separation of the tissue from the skeleton and used as reference variable for the biomass data (mg cm^{-2}).

Supplementary References

1. Pierrot, D., Lewis, E. & Wallace, D. W. R. MS Excel Program Developed for CO_2 System Calculations. (2006).
2. Naumann, M. S., Niggl, W., Laforsch, C., Glaser, C. & Wild, C. Coral surface area quantification-evaluation of established techniques by comparison with computer tomography. *Coral Reefs* **28**, 109–117 (2009).
3. Gori, A. *et al.* Physiological response of the cold-water coral *Desmophyllum dianthus* to thermal stress and ocean acidification. *PeerJ* **4**, e1606 (2016).
4. Gori, A., Reynaud, S., Orejas, C., Gili, J. M. & Ferrier-Pagès, C. Physiological performance of the cold-water corals *Dendrophyllia cornigera* reveals its preference for temperate environments. *Coral Reefs* **33**, 665–674 (2014).
