# Supplementary material for: Environmental stability and phenotypic plasticity benefit the cold-water coral Desmophyllum dianthus in an acidified fjord
Source: Commun Biol. 2022 Jul 9;5:683. doi: 10.1038/s42003-022-03622-3 (PMC9271058; doi:10.1038/s42003-022-03622-3)
Supplement: Supplementary file 3 — Description of Additional Supplementary Files [file 42003_2022_3622_MOESM3_ESM.pdf]

Description of Additional Supplementary Files

File name: Supplementary Data 1
Description: Overview of seasonal environmental conditions and carbonate chemistry in Comau Fjord, Chile. Environmental conditions at six coral stations at 20 m water depth along the fjord from head to mouth (A-F) and at one station at 300 m water depth (Ed). Water depth of coral stations is given relative to Mean Lower Low Water. Salinity and oxygen concentrations were measured with a CTD. Water samples were collected at coral stations and analysed for total alkalinity (TA) and dissolved inorganic carbon (DIC) and carbonate chemistry was calculated from TA and DIC using CO2SYS (Pierrot et al., 2006). Temperature was measured using TidbiT temperature loggers over a period of three to four months for each season. Temperature values are given as mean ± standard deviation. Temperature and salinity measurements at station X were conducted with a CTD and are given as mean ± standard deviation

File name: Supplementary Data 2
Description: Seasonal physiological parameters of native and novel *Desmophyllum dianthus* in Comau Fjord, Chile. Calcification rates, respiration rates and biomass content of *D. dianthus* (mean ± standard deviation) at six stations at 20 m water depth along the fjord from head to mouth (A-F) and at one station at 300 m water depth (Ed). Native corals were re-installed at the same station after collection in September 2016 and novel corals were cross-transplanted between the shallow stations at the head (A) and the mouth of the fjord (F), and between shallow (Es) and deep (Ed). Calcification and respiration rates were measured after four, eight and eleven months (January, May and August 2017) using the same individuals (experimental corals) in each season (N = 4-10). The tissue biomass was determined from tissue corals that were collected during each season (N = 6-10). Note that biomass data could not be obtained for all stations and seasons due to logistical problems. The tissue covered surface area was used as reference values for calcification rates (mg cm^{-2} d^{-1}) and respiration rates (µmol cm^{-2} d^{-1}) and for the tissue biomass (mg cm^{-2}). Calcification rates were also calculated in % d^{-1} using the skeletal dry mass as reference value.

File name: Supplementary Data 3
Description: Post hoc tests for calcification and respiration rates of *Desmophyllum dianthus*. Only relevant results are displayed here. Significant p-values are shown in bold.
