# Supplementary material for: Environmental stability and phenotypic plasticity benefit the cold-water coral Desmophyllum dianthus in an acidified fjord
Source: Commun Biol. 2022 Jul 9;5:683. doi: 10.1038/s42003-022-03622-3 (PMC9271058; doi:10.1038/s42003-022-03622-3)
Supplement: Supplementary file 7 — Reporting summary [file 42003_2022_3622_MOESM7_ESM.pdf]

nature portfolio

Corresponding author(s): Kristina K. Beck

Last updated by author(s): 07/06/2022

Reporting Summary

Nature Portfolio wishes to improve the reproducibility of the work that we publish. This form provides structure for consistency and transparency in reporting. For further information on Nature Portfolio policies, see our Editorial Policies and the Editorial Policy Checklist.

Statistics

For all statistical analyses, confirm that the following items are present in the figure legend, table legend, main text, or Methods section.

n/a	Confirmed	
☐	☒	The exact sample size (*n*) for each experimental group/condition, given as a discrete number and unit of measurement
☐	☒	A statement on whether measurements were taken from distinct samples or whether the same sample was measured repeatedly
☐	☒	The statistical test(s) used AND whether they are one- or two-sided<br>*Only common tests should be described solely by name; describe more complex techniques in the Methods section.*
☒	☐	A description of all covariates tested
☐	☒	A description of any assumptions or corrections, such as tests of normality and adjustment for multiple comparisons
☐	☒	A full description of the statistical parameters including central tendency (e.g. means) or other basic estimates (e.g. regression coefficient) AND variation (e.g. standard deviation) or associated estimates of uncertainty (e.g. confidence intervals)
☐	☒	For null hypothesis testing, the test statistic (e.g. *F*, *t*, *r*) with confidence intervals, effect sizes, degrees of freedom and *P* value noted<br>*Give P values as exact values whenever suitable.*
☒	☐	For Bayesian analysis, information on the choice of priors and Markov chain Monte Carlo settings
☒	☐	For hierarchical and complex designs, identification of the appropriate level for tests and full reporting of outcomes
☒	☐	Estimates of effect sizes (e.g. Cohen's *d*, Pearson's *r*), indicating how they were calculated

Our web collection on statistics for biologists contains articles on many of the points above.

Software and code

Policy information about availability of computer code

Data collection	No software was used for data collection.
Data analysis	The data were analysed with a custom-made code using the software R (version 4.1.0).

For manuscripts utilizing custom algorithms or software that are central to the research but not yet described in published literature, software must be made available to editors and reviewers. We strongly encourage code deposition in a community repository (e.g. GitHub). See the Nature Portfolio guidelines for submitting code & software for further information.

Data

Policy information about availability of data

All manuscripts must include a data availability statement. This statement should provide the following information, where applicable:

- Accession codes, unique identifiers, or web links for publicly available datasets
- A description of any restrictions on data availability
- For clinical datasets or third party data, please ensure that the statement adheres to our policy

The datasets generated and analysed during the current study are available in the data repository PANGAEA: https://doi.org/10.1594/PANGAEA.945072

Field-specific reporting

Please select the one below that is the best fit for your research. If you are not sure, read the appropriate sections before making your selection.

☒ Life sciences ☐ Behavioural & social sciences ☐ Ecological, evolutionary & environmental sciences

For a reference copy of the document with all sections, see nature.com/documents/nr-reporting-summary-flat.pdf

Life sciences study design

All studies must disclose on these points even when the disclosure is negative.

Sample size	Given the logistical and technical limitations of an in situ experiment with deep water corals in a remote location, the number of stations and corals was sufficient. We used a sample size of 8-10 corals, which is similar to other cold-water coral studies and was sufficient to address our research questions.
Data exclusions	No data were excluded from the analyses.
Replication	Due to a year round study, high number of corals (392) and the logistical complexity as well as costs, it was not possible to replicate the study temporally or spatially in another fjord. However, the seasonal evaluation of performance in situ and high number of individuals together with stations make our outcome robust.
Randomization	Corals collected in the field were of similar size and randomly assigned to experimental and tissue corals. Similarly, the assignment to native and novel corals from the same station was done randomly.
Blinding	Logistically, blinding was difficult and almost impossible for assessing in situ performance. The work included the collection and re-deployment in a remote location by divers/pulley that was done sequentially per station. However, coral tissue analyses was done blinded as corals were randomly processed and assigned numbers that could not be linked to their origin and experimental treatment.

Reporting for specific materials, systems and methods

We require information from authors about some types of materials, experimental systems and methods used in many studies. Here, indicate whether each material, system or method listed is relevant to your study. If you are not sure if a list item applies to your research, read the appropriate section before selecting a response.

Materials & experimental systems

n/a	Involved in the study	
☒	☐	Antibodies
☒	☐	Eukaryotic cell lines
☒	☐	Palaeontology and archaeology
☐	☒	Animals and other organisms
☒	☐	Human research participants
☒	☐	Clinical data
☒	☐	Dual use research of concern

Methods

n/a	Involved in the study	
☒	☐	ChIP-seq
☒	☐	Flow cytometry
☒	☐	MRI-based neuroimaging

Animals and other organisms

Policy information about studies involving animals; ARRIVE guidelines recommended for reporting animal research

Laboratory animals	The study did not involve laboratory animals.
Wild animals	The cold-water coral Desmophyllum dianthus was collected in the field (Comau Fjord, Chile) by SCUBA divers/ROV. At the end of the field experiment, the corals were snap-frozen in liquid nitrogen for further tissue and skeletal analyses and transported in dry shippers to the Alfred Wegener Institute in Germany.
Field-collected samples	The study did involve field-collected samples but only for experimental work in the field and not for maintenance under laboratory conditions.
Ethics oversight	As corals are invertebrates, no ethics oversight was required, but we followed the sampling and work guidelines established and applied at the research station in Chile to minimise impact on the corals and the surrounding environment.

Note that full information on the approval of the study protocol must also be provided in the manuscript.
